# Oral Antimicrobial Peptides and Biological Control of Caries

**DOI:** 10.1186/1472-6831-6-S1-S13

**Published:** 2006-06-15

**Authors:** Beverly A Dale, Renchuan Tao, Janet R Kimball, Richard J Jurevic

**Affiliations:** 1 Dept. of Oral Biology, Box 357132, University of Washington, Seattle WA 98195, USA; 2 Guangxi Medical University, Nanning, Guangxi, P.R.China; 3 Dept. of Oral Diagnosis, University of Illinois at Chicago, Chicago, IL, USA

## Abstract

The presence of antimicrobial peptides (AMPs) in saliva may be a biological factor that contributes to susceptibility or resistance to caries. This manuscript will review AMPs in saliva, consider their antimicrobial and immunomodulatory functions, and evaluate their potential role in the oral cavity for protection of the tooth surface as well as the oral mucosa. These AMPs are made in salivary gland and duct cells and have broad antimicrobial activity. Alpha-defensins and LL37 are also released by neutrophils into the gingival crevicular fluid. Both sources may account for their presence in saliva. A recent study in middle school children aimed to determine a possible correlation between caries prevalence in children and salivary concentrations of the antimicrobial peptides human beta-defensin-3 (hBD-3), the cathelicidin, LL37, and the alpha-defensins. The levels of these AMPs were highly variable in the population. While levels of LL37 and hBD-3 did not correlate with caries experience, the mean alpha-defensin level was significantly higher in children with no caries than in children with caries (p < 0.005). We conclude that several types of AMPs that may have a role in oral health are present in unstimulated saliva. Low salivary levels of alpha-defensin may represent a biological factor that contributes to caries susceptibility. Our observation could lead to new ways to prevent caries and to a new tool for caries risk assessment.

## Introduction

**Figure 1:**
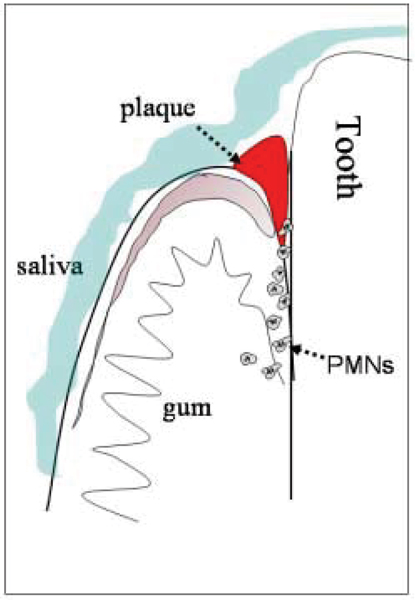
Diagram indicating the presence of antimicrobial peptides in saliva (blue), in gingival epithelium (pink), and in neutrophils (PMNs) migrating into the oral cavity via the gingival crevicular fluid. All of these sources of antimicrobial peptides may aid protection of the mucosa from bacterial infection and the tooth surface from caries.

The oral cavity is a unique environment. Oral mucosae are a critical protective interface between external and internal environments and must serve as a barrier to the myriad microbial species present in the mouth. Saliva, the epithelial surface layers, and polymorphonuclear leukocytes (neutrophils) all contribute to maintaining the health of the oral cavity and periodontium in overlapping but independent ways (Figure 1). Antimicrobial peptides (AMPs) are natural antibiotics that are found in each of these compartments: in the saliva, in the epithelium, and in neutrophils. Evidence is accumulating that AMPs are important contributors to maintaining the balance between health and disease as part of the host innate immune response. They have generally been considered to contribute to mucosal health; however, it is logical that these AMPs are biological factors that influence caries susceptibility and development as well.

This manuscript will review oral AMPs, consider their role as genetically inherited factors that may be among the biological controls that influence caries risk, briefly describe a study of AMPs in children with and without caries, and discuss the potential utility of one particular AMP in caries risk assessment and prevention.

## Oral AMPs and caries

Oral AMPs provide a first line of defense against a wide spectrum of pathogens [1-3]. Members of the three main AMP families are found in the oral cavity. These are defined by biochemical and structural characteristics: 1) α-helical peptides without cysteine (the cathelicidin, LL37) [4]; 2) peptides with three disulphide bonds (the α- and β- defensins) [1,5]; and 3) peptides with an unusually high proportion of specific amino acids; for example, the histatins [6].

Recent research suggests the importance of the defensins and the cathelicidin LL37 as antibacterial agents in the oral cavity [7], while histatins are primarily antifungal agents [6]. The human β-defensins (hBDs) are widely expressed in oral tissues including gingival epithelium [8,9], salivary glands and ducts and saliva [10,11]. The neutrophil alpha-defensins, (human neutrophil peptides 1–3 (HNP1–3)), are one of the mechanisms for non-oxidative microbial killing [5] and are found in gingival crevicular fluid [12]. The human cathelicidin peptide, LL37, is in neutrophils, inflamed epithelia, submandibular salivary glands and saliva [13,14].

With antimicrobial peptides now strongly implicated in the host innate immune response, in particular in the oral cavity (reviewed by Dale and Fredericks, Ganz) [7,1], their availability in unstimulated saliva implies their potential role in protecting tooth structure from bacterially-induced caries, either by direct killing or by prevention of biofilm formation on the tooth surface.

The defensins and cathelicidin have broad antimicrobial activity against gram-negative and gram-positive bacteria and are effective against oral microorganisms such as S*treptococcus mutans, Porphyromonas gingivalis *and *Actinobacillus actinomycetemcomitans *[7,15-19]. The expression and major activities of AMPs in saliva are summarized in Table 1; however it should be noted that these peptides have both species and strain specificity, for unknown reasons, and it is difficult to generalize their antimicrobial function. The cathelicidins and defensins act synergistically with other antimicrobials [20,21]. Thus, the co-expression in saliva of LL37 and defensins with peptides such as histatin, proline-rich proteins, and calprotectin may provide a natural antibiotic barrier.

## Genetic influences on AMP expression and on caries

The amount of AMPs expressed in saliva varies between individuals. This has been previously demonstrated for alpha- and beta-defensins, histatin, and proline-rich proteins [6,22,23]. Our initial studies in children show that the levels of these peptides in unstimulated saliva vary greatly between individuals, even when differences in total salivary protein are considered (see below). The large variation in concentration of defensins in saliva and in their mRNA in oral tissues could be attributed to the organization of their genes. The genes for the alpha- and beta-defensins lie in a cluster on human chromosome 8. Several genes in this region can occur as multiple repeated copies. This is known as copy number polymorphism [24,25]. We do not yet know if individuals with multiple copies of a particular gene, for example, HNP1, make more of the protein, but this seems quite likely. In other words, individual differences in the amount of alpha- and beta-defensins may be genetically determined. In contrast, there is only one copy of the LL37 gene, which is located on chromosome 3.

There is also evidence for genetically determined factors in susceptibility to caries. Anecdotal evidence suggests individual differences in caries experience in children even within the same family [26]. These individual differences suggest that biological factors, which may be genetically determined, have a role in caries resistance and susceptibility. Studies of twins offer the strongest evidence to date for the importance of biologically inherited factors contributing to caries susceptibility [27-29]. Twins show significantly greater similarity of caries experience in monozygotic (MZ, identical) than in dizygotic (DZ, fraternal) twins (reviewed by Shuler) [30].

## Salivary proteins and caries experience

Salivary constituents are potential candidates as biological factors influencing caries risk. Many salivary protein components, such as proline-rich glycoprotein, mucins, immunoglobulins, agglutinin, lactoferrin, cystatins and lysozyme are thought to have a role in defense in the oral cavity [31]. Numerous studies have investigated the correlation among these salivary proteins and glycoproteins and caries experience, but no studies have shown reliable association between a single salivary component and caries experience [32,33]. **The expression of AMPs in saliva and throughout the oral cavity suggests that they may have a role in protecting tooth structure from caries as well as protecting oral mucosa**. Several reasons for this proposal are 1) AMPs have broad antimicrobial activity; 2) their action is synergystic with other antimicrobials in saliva; co-expression of cathelicidins and defensins with peptides such as histatin, proline-rich proteins may enhance antimicrobial function; 3) they stimulate the acquired immune system and could function to enhance IgA production as well as IgG production [2]; 4) these AMPs may function to keep overall bacteria in check and to help prevent biofilm formation. Thus, oral AMPs may provide a natural antibiotic barrier.

## AMPs and caries in middle school children

To investigate the possible relationship between AMP levels in saliva and caries experience, we conducted a study in middle school children located in a rural area in Washington state [34]. One hundred and forty-nine children (88 females, 61 males) participated in the study. All children were between 11 and 15 years of age. Most of the population was Hispanic with some Native Americans and Caucasians. A brief health history survey was completed by parents of the subjects. Oral examinations were performed by trained calibrated clinicians using standardized procedures. Overall, the children were healthy with 92% having no history of major illness or disease. The study was conducted with permission of school officials and informed consent of subjects and parents obtained through an educational session and written bilingual consent in accordance with a protocol approved by the University of Washington Institutional Review Board. A brief health history was taken, and unstimulated saliva samples collected and stored in a frozen state for later analysis. Examiners were instructed to rank subjects separately for active caries and for filled surfaces as 0, no decayed or filled surfaces; 1, mild (1–2 affected surfaces); 2, moderate (3–6 affected surfaces); and 3, severe (>6 affected surfaces). Final caries experience score was determined as the sum of the scores for active decay and filled surfaces. Fifty-three subjects (36%) had no decay, 37 (24%), 39 (27%), and 20 (13%) had caries scores of 1, 2, and 3 or greater, respectively.

For analysis, saliva samples were thawed and cleared by centrifugation twice at 15,000 rpm for 10 min. Cleared saliva samples were assayed for total protein concentration by the BCA assay (Pierce Inc., Rockford, IL, USA) and for alpha-defensin HNP1–3 ELISA assay according to manufacturer's instructions (HyCult Biotechnology, Uden, Netherlands). This assay measures HNP1, 2, and 3 because the antibody does not distinguish between these closely related peptides. Aliquots of cleared saliva were acid-extracted for assay of LL37 and hBD3 by slot immunoblot analysis (LL37 by assay from Phoenix Pharmaceuticals Inc., Belmont, CA and hBD3 using polyclonal antibody from Orbigen Inc, San Diego, CA). The mean protein concentration of unstimulated saliva samples was 1633 ± 908 μg/ml (range from 421 to 7052 μg/ml). This value agrees with previously reported total protein concentration for this age group [35]. The salivary protein concentration showed no correlation with age, gender, or caries score. Antimicrobial peptide (AMP) concentrations were in the μg/ml range. AMP levels were also normalized to the protein concentration in whole saliva for each sample. HNP1–3, hBD3 and LL37 all showed extensive variation in concentration in our population, even when normalized to total salivary protein levels. Mean values for HNP1–3, hBD3 and LL37 were 0.94, 0.73, 3.36 μg/ml, respectively, with the range extending 10-fold up and down from the mean. The concentration of AMPs in unstimulated saliva of children has not been previously reported, although normal adults have a mean value of 0.8 μg/ml HNP [36].

**Figure 2:**
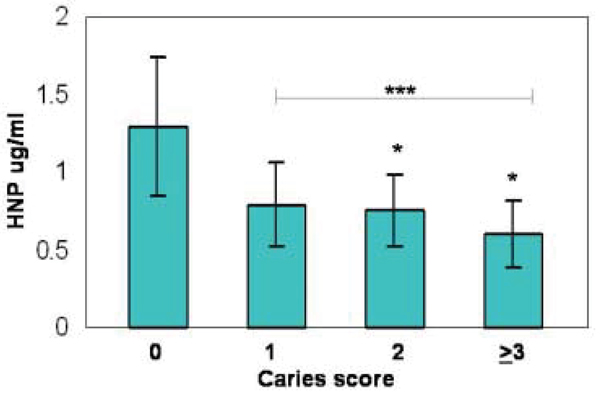
HNP 1–3 concentration in saliva as a function of caries score. The HNP1–3 peptides are closely related and are all detected by the immunoassay used. Note statistically significant difference between the caries groups and the 0 Caries group; * p < 0.05; *** p < 0.005.

When the relationship of AMP expression and caries experience was evaluated, we found a significant difference in the level of HNP1–3 among different caries groups (p < 0.005). Differences were observed for both the mean (or median) level of salivary HNP1–3 concentration (μg/ml) and salivary HNP1–3 relative to salivary protein (μg/mg). The HNP1–3 concentration was 1.30 ± 0.22 μg/ml (median ± std. error of the mean) for the caries free group (n = 51) and 0.73 ± 0.07 μg/ml for all subjects with evidence of caries (n = 92) (independent sample two tailed T test, p = 0.005). The HNP1–3 value relative to total salivary protein was 0.84 ± 0.14 μg/mg protein in the caries free group and 0.48 ± 0.05 μg/mg protein in the combined caries group (p = 0.005) (Figure 2). Similar analysis for LL37 showed the same trend with higher levels of LL37 in the no caries group than in those with caries, but results were not statistically significant. HBD3 concentration in saliva and the level of hBD3 relative to protein showed no significant difference in our population or between the different caries groups (not shown). Statistical analyses were also done using the Kruskal-Wallis non-parametric test based on rank and designed for non-normally distributed data. Using this test, significance differences were verified or even improved. We also evaluated whether association with caries could be due to *either *HNP1–3 *or *LL37, but over 90% of the effect was due to HNP1–3.

Finally, to further examine the relationship of HNP1–3 with caries, the HNP concentration values were divided into quartiles and evaluated for subjects with no caries compared to those with caries (Table 2). An increasing proportion of subjects had no caries as the HNP concentration increased; only 14% of the subjects with HNP1–3 levels of < 0.4 μg/ml (n = 36) were caries free, but 55% of the subjects with HNP1–3 levels greater than 1.08 μg/ml (n = 36) were caries free. It can be readily seen that children with low salivary alpha-defensin have greater levels of caries.

There were two major findings from our study. First, there was extensive variation in AMP levels between individuals; levels varied by 100 fold from the lowest to highest even when adjusted for total salivary protein. Second, the level of alpha-defensins HNP1–3 was inversely correlated with caries (Figure 2). Our findings suggest that low salivary levels of HNP1–3 may contribute to caries susceptibility and could be a new and useful measure of the risk for caries in children.

## Where do the salivary HNPs and other AMPs come from?

The most obvious source for salivary AMPs is secretion by salivary gland and duct cells. LL-37 and beta-defensins are expressed in salivary glands [7,8,10,13,14]. In addition, we have shown HNP1–3 by immunohistochemistry in submandibular salivary duct cells [34]. The submandibular glands are the major source of unstimulated saliva, suggesting that these cells may be a source of AMPs in saliva. However, an important alternative source for HNP1–3 and LL37 are the neutrophils that migrate into the oral cavity via gingival crevicular fluid. In normal individuals it has been estimated that 30,000 neutrophils per minute enter the oral cavity via this route through the junctional epithelium surrounding the teeth [37]. This flow of neutrophils is required for periodontal health, and defects in neutrophil function and chemotatsis are associated with early onset periodontal disease in children [38,39]. Our findings suggest that this flow of neutrophils may also be important for protection from caries.

## How does HNP provide protection?

HNP1–3 in saliva could contribute to resistance to caries by direct antimicrobial properties (either alone or in combination with other saliva components) or by preventing biofilm formation on the surface of the tooth via its ability to bind bacterial outer membranes. The AMP levels found in saliva in this study are in the range of effective antimicrobial function for beta-defensins vs. *S. mutans *[17] although effectiveness of HNPs against *S. mutans *has not been reported. The low ionic strength in saliva is conducive to antimicrobial activity and thus may impact the flora of the oral cavity and exert a beneficial effect on dental health. In addition, alpha- and beta-defensins as well as LL37 have other immunomodulatory and chemoattractant effects, and individuals with high expression may benefit from these effects. The inverse correlation of HNP1–3 with caries experience suggests its possible protective effect. Conversely, low levels of HNP1–3 may result in increased susceptibility to caries.

## Conclusion

We have many follow-up questions. Based on our study design, we cannot yet determine if the level of salivary alpha-defensin is predictive of future caries, but we have shown that children with caries have significantly lower levels of alpha-defensins based both on their concentration in saliva and their relationship to total salivary protein. Can this finding be replicated in younger children? This is important because of the need for new approaches for caries risk assessment in young children, when preventive measures are likely to have the greatest impact. Identifying those children who are at highest risk for disease will enable limited resources to be targeted toward individualized, aggressive and pro-active interventions to prevent caries. Can the level of AMPs be replicated over time? Could alpha-defensins be used therapeutically or in a prospective manner in mouthwash or toothpaste or chewing gum? Is the effect primarily an antimicrobial effect, or is it also due to immunomodulatory functions of these peptides? Do children with very low alpha-defensin levels have different flora than children with high alpha-defensin levels? Is there a relationship between the intensity of tooth brushing and levels of salivary AMPs? Perhaps vigorous brushing could stimulate outflow of the neutrophils via the gingival sulcus. In addition, the junctional epithelium is innervated with substance P expressing nerve fibers. Irritation provoked by vigorous brushing might stimulate local release of this neuropeptide, which in turn has profound chemoattractive activity for neutrophils [40,41].

The prediction of caries risk has been of long-standing interest and is very important for development of new preventive strategies. This is especially significant for young children and for children with special health care needs. Saliva is an easily available fluid which can be collected non-invasively and used to measure and monitor the risk for caries [42]. The assay for HNP1–3 is easy to perform and can be done using less than 0.2 ml crude whole saliva. Low salivary levels of alpha-defensins (HNP1–3) could be a new and useful measure of the risk for caries in children. Future studies could lead to development of means to enhance endogenous oral peptide expression, to utilization of these peptides as therapeutics, and to the development of a simple test for clinical evaluation of caries risk.

## Competing interests

The author(s) declare that they have no competing interests.

## Authors' contributions

All authors read and approved the final manuscript.

## Figures and Tables

**Table 1:** Antimicrobial Peptides in Saliva

**Peptide**	**Origin**	**Role/comments**	**Major Oral Microbial Targets**	**References**
Alpha-defensins HNP 1–4	Neutrophils, gingival sulcus, sites of inflammation, salivary duct cells	Antibacterial, antifungal, antiviral. Present in GCF	*Candida albicans HIV S. mutans *(poor)	(Raj *et al*., 2000; Zhang *et al*., 2002) [43, 44]
LL-37	Neutrophils, gingival sulcus, salivary glands and ducts	Primarily antibacterial	*S. mutans F. nucleatum A. actinomycetemcomitans Capnocytophaga sputigena*	(Ouhara *et al*., 2005; Tanaka *et al*., 2000) [17, 19]
Beta-defensins hBD1 hBD2 hBD3	Epithelia, salivary ducts	Antibacterial, antifungal, antiviral. Part of the protective barrier function of epithelium. Secreted	hBD1: Poor antibacterial *hBD2, hBD3: S. mutans S. sanguis F. nucleatum P. gingivalis Candida albicans *HIV	(Joly *et al*., 2004; Maisetta *et al*., 2003; Quinones-Mateu *et al*., 2003)[15, 20, 18]
Histatins	Salivary glands/ducts	Antifungal	*Candida albicans*	(Oppenheim *et al*., 1988) [6]

**Table 2:** Caries Free Children by HNP score

**HNP μg/ml**	**n**	**Caries Free (n)**	**Caries Free (%)**
0.06–0.39	36	5	14%
0.40–0.60	36	12	33%
0.60–1.03	36	14	39%
1.08–10.5	36	20	55%
			
Total pop.	144	51	35%

## References

[B1] Ganz T (2003). Defensins: antimicrobial peptides of innate immunity. Nat Rev Immunol.

[B2] Yang D, Biragyn A, Hoover DM, Lubkowski J, Oppenheim JJ (2004). Multiple Roles of Antimicrobial Defensins, Cathelicidins, and Eosinophil-Derived Neurotoxin in Host Defense. Annu Rev Immunol.

[B3] Zasloff M (2002). Antimicrobial peptides of multicellular organisms. Nature.

[B4] Bals R, Wilson JM (2003). Cathelicidins – a family of multifunctional antimicrobial peptides. Cell Mol Life Sci.

[B5] Ganz T, Selsted ME, Szklarek D, Harwig SS, Daher K, Bainton DF, Lehrer RI (1985). Defensins. Natural peptide antibiotics of human neutrophils. J Clin Invest.

[B6] Oppenheim FG, Xu T, McMillian FM, Levitz SM, Diamond RD, Offner GD, Troxler RF (1988). Histatins, a novel family of histidine-rich proteins in human parotid secretion. Isolation, characterization, primary structure, and fungistatic effects on Candida albicans. J Biol Chem.

[B7] Dale BA, Fredericks LP (2005). Antimicrobial peptides in the oral environment: expression and function in health and disease. Curr Issues Mol Biol.

[B8] Dunsche A, Acil Y, Dommisch H, Siebert R, Schroder JM, Jepsen (2002). The novel human beta-defensin-3 is widely expressed in oral tissues. Eur J Oral Sci.

[B9] Zhao C, Wang I, Lehrer RI (1996). Widespread expression of beta-defensin hBD-1 in human secretory glands and epithelial cells. FEBS Lett.

[B10] Bonass WA, High AS, Owen PJ, Devine DA (1999). Expression of beta-defensin genes by human salivary glands. Oral Microbiol Immunol.

[B11] Sahasrabudhe KS, Kimball JR, Morton T, Weinberg A, Dale BA (2000). Expression of the antimicrobial peptide, human b-defensin 1, in duct cells of minor salivary glands and detection in saliva. J Dent Res.

[B12] McKay MS, Olson E, Hesla MA, Panyutich A, Ganz T, Perkins S, Rossomando EF (1999). Immunomagnetic recovery of human neutrophil defensins from the human gingival crevice. Oral Microbiol Immunol.

[B13] Murakami M, Ohtake T, Dorschner RA, Gallo RL (2002). Cathelicidin antimicrobial peptides are expressed in salivary glands and saliva. J Dent Res.

[B14] Woo JS, Jeong JY, Hwang YJ, Chae SW, Hwang SJ, Lee HM (2003). Expression of cathelicidin in human salivary glands. Arch Otolaryngol Head Neck Surg.

[B15] Joly S, Maze C, McCray PB, Guthmiller JM (2004). Human beta-defensins 2 and 3 demonstrate strain-selective activity against oral microorganisms. J Clin Microbiol.

[B16] Nishimura E, Eto A, Kato M, Hashizume S, Imai S, Nisizawa T, Hanada N (2004). Oral streptococci exhibit diverse susceptibility to human beta-defensin-2: antimicrobial effects of hBD-2 on oral streptococci. Curr Microbiol.

[B17] Ouhara K, Komatsuzawa H, Yamada S, Shiba H, Fujiwara T, Ohara M, Sayama K, Hashimoto K, Kurihara H, Sugai M (2005). Susceptibilities of periodontopathogenic and cariogenic bacteria to antibacterial peptides, {beta}-defensins and LL37, produced by human epithelial cells. J Antimicrob Chemother.

[B18] Quinones-Mateu ME, Lederman MM, Feng Z, Chakraborty B, Weber J, Rangel HR, Marotta ML, Mirza M, Jiang B, Kiser P, Medvik K, Sieg SF, Weinberg A (2003). Human epithelial beta-defensins 2 and 3 inhibit HIV-1 replication. AIDS.

[B19] Tanaka D, Miyasaki KT, Lehrer RI (2000). Sensitivity of Actinobacillus actinomycetemcomitans and Capnocytophaga spp. to the bactericidal action of LL-37: a cathelicidin found in human leukocytes and epithelium. Oral Microbiol Immunol.

[B20] Maisetta G, Batoni G, Esin S, Luperini F, Pardini M, Bottai D, Florio W, Giuca MR, Gabriele M, Campa M (2003). Activity of human beta-defensin 3 alone or combined with other antimicrobial agents against oral bacteria. Antimicrob Agents Chemother.

[B21] Nagaoka I, Hirota S, Yomogida S, Ohwada A, Hirata M (2000). Synergistic actions of antibacterial neutrophil defensins and cathelicidins. Inflamm Res.

[B22] Ayad M, Van Wuyckhuyse BC, Minaguchi K, Raubertas RF, Bedi GS, Billings RJ, Bowen WH, Tabak LA (2000). The association of basic proline-rich peptides from human parotid gland secretions with caries experience. J Dent Res.

[B23] Tanida T, Okamoto T, Okamoto A, Wang H, Hamada T, Ueta E, Osaki T (2003). Decreased excretion of antimicrobial proteins and peptides in saliva of patients with oral candidiasis. J Oral Pathol Med.

[B24] Hollox EJ, Armour JA, Barber JC (2003). Extensive normal copy number variation of a beta-defensin antimicrobial-gene cluster. Am J Hum Genet.

[B25] Mars WM, Patmasiriwat P, Maity T, Huff V, Weil MM, Saunders GF (1995). Inheritance of unequal numbers of the genes encoding the human neutrophil defensins HP-1 and HP-3. J Biol Chem.

[B26] Filstrup SL, Briskie D, da Fonseca M, Lawrence L, Wandera A, Inglehart MR (2003). Early childhood caries and quality of life: child and parent perspectives. Pediatr Dent.

[B27] Boraas JC, Messer LB, Till MJ (1988). A genetic contribution to dental caries, occlusion, and morphology as demonstrated by twins reared apart. J Dent Res.

[B28] Conry JP, Messer LB, Boraas JC, Aeppli DP, Bouchard TJ (1993). Dental caries and treatment characteristics in human twins reared apart. Arch Oral Biol.

[B29] Hicks J, Garcia-Godoy F, Flaitz C (2003). Biological factors in dental caries: role of saliva and dental plaque in the dynamic process of demineralization and remineralization (part 1). J Clin Pediatr Dent.

[B30] Shuler CF (2001). Inherited risks for susceptibility to dental caries. J Dent Educ.

[B31] Van Nieuw Amerongen A, Bolscher JG, Veerman EC (2004). Salivary proteins: protective and diagnostic value in cariology?. Caries Res.

[B32] Kirstila V, Hakkinen P, Jentsch H, Vilja P, Tenovuo J (1998). Longitudinal analysis of the association of human salivary antimicrobial agents with caries increment and cariogenic micro-organisms: a two-year cohort study. J Dent Res.

[B33] Tenovuo J, Jentsch H, Soukka T, Karhuvaara L (1992). Antimicrobial factors of saliva in relation to dental caries and salivary levels of mutans streptococci. J Biol Buccale.

[B34] Tao R, Jurevic RJ, Coulton KK, Tsutsui MT, Roberts MC, Kimball JR, Wells NJ, Berndt J, Dale BA (2005). Salivary antimicrobial peptide expression and dental caries experience in children. Antimicrob Agents Chemother.

[B35] Ben-Aryeh H, Fisher M, Szargel R, Laufer D (1990). Composition of whole unstimulated saliva of healthy children: changes with age. Arch Oral Biol.

[B36] Mizukawa N, Sugiyama K, Ueno T, Mishima K, Takagi S, Sugahara T (1999). Defensin-1, an antimicrobial peptide present in the saliva of patients with oral diseases. Oral Dis.

[B37] Schroeder HE (1986). The Periodontium.

[B38] Page RC, Vandesteen GE, Ebersole JL, Williams BL, Dixon IL, Altman LC (1985). Clinical and laboratory studies of a family with a high prevalence of juvenile periodontitis. J Periodontol.

[B39] Putsep K, Carlsson G, Boman HG, Andersson M (2002). Deficiency of antibacterial peptides in patients with morbus Kostmann: an observation study. Lancet.

[B40] Brogden KA, Guthmiller JM, Salzet M, Zasloff M (2005). The nervous system and innate immunity: the neuropeptide connection. Nat Immunol.

[B41] Tanaka T, Kido MA, Ibuki T, Yamaza T, Kondo T, Nagata E (1996). Immunocytochemical study of nerve fibers containing substance P in the junctional epithelium of rats. J Periodontal Res.

[B42] Streckfus CF, Bigler LR (2002). Saliva as a diagnostic fluid. Oral Dis.

[B43] Raj PA, Antonyraj KJ, Karunakaran T (2000). Large-scale synthesis and functional elements for the antimicrobial activity of defensins. Biochem J.

[B44] Zhang L, Yu W, He T, Yu J, Caffrey RE, Dalmasso EA, Fu S, Pham T, Mei J, Ho JJ, Zhang W, Lopez P, Ho DD (2002). Contribution of human alpha-defensin 1, 2, and 3 to the anti-HIV-1 activity of CD8 antiviral factor. Science.

